# Antioxidant and Anti-Inflammatory Activities of the Crude Extracts of Raw and Fermented Tomato Pomace and Their Correlations with Aglycate-Polyphenols

**DOI:** 10.3390/antiox9020179

**Published:** 2020-02-21

**Authors:** Parisa Abbasi-Parizad, Patrizia De Nisi, Fabrizio Adani, Tommy Pepé Sciarria, Pietro Squillace, Alessio Scarafoni, Stefania Iametti, Barbara Scaglia

**Affiliations:** 1 Ricicla Group Labs-Department of Agricultural and Environmental Sciences (DISAA), University of Milan, via Giovanni Celoria 2, 20133 Milan, Italy; parisa.abbasi@unimi.it (P.A.-P.); patrizia.denisi@unimi.it (P.D.N.); fabrizio.adani@unimi.it (F.A.); tommy.pepe@unimi.it (T.P.S.); pietro.squillace@unimi.it (P.S.); 2 Department of Food, Environmental and Nutritional Sciences (DeFENS), University of Milan, via Giovanni Celoria 2, 20133 Milan, Italy; alessio.scarafoni@unimi.it (A.S.); stefania.iametti@unimi.it (S.I.)

**Keywords:** lactic fermentation, tomato pomace, Partial Least Square, polyphenols, anti-inflammatory

## Abstract

Two tomato pomace (TP) were studied as feedstocks to obtain extracts that are rich in polyphenols. TPs prompt degradation impairs biomass safety, thus naturally present microflora were tested to perform conservation, and own lactic bacteria became predominant after 60 days of treatment. The extracts of TPs and TPs fermented (TPF) were chemically characterized and tested for antioxidant and anti-inflammatory activities. Flavonoids and phenolic acids were classed as aglycone-polyphenols (A-PP), the most bioactive polyphenol fraction. Fermentation led to a reduction of the A-PP amount, but no significant change in composition. Antioxidant power increased, despite the A-PP reduction, for the presence of fermentation metabolites having aromatic-substituent. TP and TPF both have anti-inflammatory properties that were strictly dependent upon the A-PP content. Fermentation preserved the anti-inflammatory activity and the Partial Least Square (PLS) identified as the most active molecules naringenin chalcone, kaempferol, gallic acid, and cinnamic acid, together with the definition of the active dose.

## 1. Introduction

Polyphenols are widely known for their positive effects on health, including anti-oxidant activity, anti-inflammatory, anti-diabetic, anti-obesity, anti-microbial, anti-proliferation, anti-allergic properties, and the prevention of chronic disease [1]. Polyphenols are of interest for the nutraceutical, cosmetic, and food sectors and they have a Global Market valued at $757 million in 2015 supported by a Compound Annual Growth Rate (CAGR) of 8.26% during the forecast period 2014–2022, based on the evidence of their activity [2]. The functional food and functional beverage segments will make major contributions to the supply of polyphenols and the dietary supplement segment is also expected to have stable growth during the forecast period, which is supported by increases in the geriatric populations in different countries [2].

Apples, green tea, and grape seeds are actually the major polyphenol feedstocks; however, different agro-food wastes have recently been tested with the aim of valorizing existing food waste substances and developing a circular economy production system [3]. 

The tomato transformation industry is a relevant sector that transforms 40 million tons in California, China and the European Union (EU) [4]. Tomatoes are mainly processed into juice, sauces, and pastes, generating huge amounts of tomato peels and seeds, named tomato pomace (TP), which is the most abundant solid waste fraction, together with other wastes, such as discarded fruit, liquid waste, sludge, etc. [5]. Being composed of skin and seeds, TP has an important content of bioactive molecules. Until recently, attention mainly focused on the characterization and extraction of lycopene, a very powerful antioxidant carotenoid that is present in the tomato pulp, but above all in the skin, which has a protective role for human health [1]. Additional antioxidant molecules have been found in the TP: for example, tocopherol and phytosterol, which are often extracted with lycopene and other hydrophilic antioxidant molecules, such as flavonoids, phenolic acids, and vitamins, which are all hydrophilic.

The tomato skin concentrates approximately 98% of tomato polyphenols [6] that are usually not degraded during industrial processes. Flavonoids and phenolic acids are the most abundant classes, with concentrations that depend on the tomato typology and cultivation conditions [6].

TP is subject to fast biological attacks that affect the safety state of the biomass due to the high moisture content and its easily biodegradable nature. To avoid damage, lactic fermentation was tentatively applied as a storage system, taking advantage of the lactic bacteria (LAB) naturally present in the tomato biomass [7]. LAB metabolism converted the available sugars into lactic acid and created very acidic conditions that were not compatible with pathogen microorganisms’ survival. The fermentation was recently tested alone or in combination with enzymes as a pretreatment to improve the extraction and bioactivity of polyphenols and vitamins [8].

This positive effect on polyphenols’ availability was linked to the LAB fiber consumption that converts polyphenol-bound into free polyphenols (A-PP), dimers, sulfo-conjugates, glucuronides, and other forms, thus increasing their extractability [9,10].

Antioxidant power is the best known polyphenol activity, assessed as being chemically reactive against radical and oxidant agents [11]. The results of those tests were useful for obtaining bioactivity screening data, although did not identify the specific physiological effects (i.e., anti-mutagenic, antiplatelet, ACE inhibitory properties, anti-inflammatory, anti-allergic, etc.) on which polyphenols act [3,12].

Several phenolic compounds, individually or in combination with other molecules, were tested on Caco-2 cells and their anti-inflammatory properties were correlated to chemical structure and dose, and molecules’ chemical interactions were determined (synergism, additive, or antagonistic) [13]. The same approach was considered to be unfeasible for the vegetal extracts complex from a composition point of view [14].

However, the possibility to identify the importance of single or a few components is an interesting topic in the perspective of imitating the molecules based on the qualitative and quantity profiles. The Partial Least Square (PLS) method has been recently applied as a screening tool to link the chemical composition of vegetal extracts to specific bioactivity [15].

This chemometric method was able to identify: (i) molecules with more activity in a mixture, (ii) synergistic and antagonistic effects when testing vegetal extracts as fingerprints vs. bioactivity [16,17].

To the best of our knowledge, no reports on the anti-inflammatory effects of TP phenolic components are available. The aim of this study was to characterize the polyphenols’ profiles from two tomato pomace (TP) sources that were sampled from the tomato canning industry, both raw and fermented, and to evaluate anti-oxidants and assess in vitro anti-inflammatory capacities while using chemical and biological tests. In addition, a first attempt was made to identify the most active compounds among the A-PP molecules and their anti-inflammatory activity to attribute more precise prominence to the different molecules and investigate the mixture effect by applying PLS.

## 2. Materials and Methods 

### 2.1. Materials

Tomato pomace from two different tomato industry (TP1 and TP2) was collected during July 2018 to September 2018 in the north of Italy from a tomato processing industry (OPOE facility in Dodici Morelli Ferrara, Emilia Romagna Region, Italy). Each biomass (10 kg) was sampled to execute the analytical characterization. TP1 and TP2 were successively dried in a vacuum oven (at 50 °C for 300 min) to measure dry matter (d.m.) content [18]. Thus, the dried samples were ground in a laboratory mill (Thomas Wiley, Thomas Scientific, Swedesboro, NJ, USA) until obtaining a very fine powder. The samples were stored at 4 °C and in darkness until further analyses. Moreover fibre analyses were performed with the aim of measuring the cell soluble (CS), cellulose, hemicellulose, and acid detergent lignin (ADL) content [18]. 

### 2.2. Tests of Fermentation and Analytical Characterization

Approximately 300 g of TP1 and TP2 wet weight (*w.w.*) were packed into airtight glass containers of 500 mL and pressed to favor the air exit, and then TP were fluxed with N_2_ before being closed. The containers were stored at 20 °C in dark conditions for 240 days; the samples were collected after 20, 30, 60, 105, and 240 days. The pH was assessed on TP according to the analytical method that was established for wastewater sludge and digested samples [19]. Organic acids concentration and speciation were determined by using a Shimadzu high-pressure liquid chromatograph (HPLC), (Shimadzu corporation, Tokyo, Japan), that was equipped with a Hi-Plex H Agilent column (300 × 7.7 mm, PL1170-6830) (Agilent technologies, Santa Clara, CA, USA), 20 µL of sample were injected with an autosampler, and the column temperature was kept at 50 °C; the H_2_SO_4_ mobile phase flow was isocratic at 0.4 mL/min and the duration of each run was 60 min Chromatograms were integrated with a Labsolution 5.90 software Shimadzu Corporation, Tokyo, Japan). All of the analyses were performed in triplicate. At the end of the fermentation, samples TP1F and TP2F i.e., TP1 and TP2 after 240 d of fermentation, respectively, were prepared to be subsequently analyzed.

### 2.3. Total Phenolic Extraction and Quantification 

Polyphenols of TP1, TP2, TP1F, and TP2F were extracted by applying the method that was reported by Valdez-Morales et al. [12]. In brief, approximately 0.5 g of finely ground TP flour were suspended in 5 mL of methanol and placed on a shaker during the extracting time. The samples were sonicated for 1 h (with shaking every 10 min), and were then transferred to a 50 mL dark bottle, adding methanol (Pro. N. 34860, Merck, Sigma Aldrich, Dermstadt, Germany) that was made up to 20 mL, and then were left in incubate overnight (25 °C/darkness/220 rpm, 22 h). The sample was subsequently centrifuged at 1500 g for 3 min to separate the polyphenol fraction solution from the TP residue, which was then re-extracted while applying the procedure that was described one time more. The supernatants from both steps were collected together and were concentrated to dryness at 37 °C in a rotary evaporator (Yamato, Santa Clara, CA, USA) [12]. The extracted TP was then re-suspended by adding 3 mL of methanol and then filtered through 0.22 μm (Durapore syringe filters Millipore, Carrigtwohill, Cork, Ireland) for the later analytical determinations.

The total phenolic content (TPC) was measured by applying the Folin–Ciocalteu reagent method with some modification [12], by diluting the crude extracts in methanol (1:30 *v*/*v*). 1 mL of 0.5 N Folin−Ciocalteu’s phenol reagent was added to 100 μL of the sample solution followed by the addition of 2 mL of Na_2_CO_3_ 20% (*w*/*v*) solution after 3 min; the mixture was left at room temperature for 15 min in darkness, and the absorbance was measured at 734 nm in a spectrophotometer UV/VIS Cary 60 (Agilent Technologies, Santa Clara, CA, USA). A calibration curve was prepared with gallic acid (GA) (G7384, Sigma Aldrich, Dermstadt, Germany) and the results were reported as milligrams of gallic acid equivalents per gram of TP samples.

### 2.4. Profiling Aglycone-Polyphenol Compounds (A-PP) by HPLC

Next, the TP extracts were characterized while using HPLC (Agilent Technologies 1260 Infinity system, Santa Clara, CA, USA). Compounds separation was conducted using a Kinetex column (C18, 5 μm, 120 Å, 4.6 × 250 mm, Phenomenex Torrance, Santa Clara, CA, USA), at 25 °C with a UV detection (Valdez-Morales et al., 2014), the flow rate was 0.5 mL min^−1^, and the injection volume was 20 μL. The compounds’ elution was done with a gradient of two solvents: acidified water with acetic acid (Pro. N. 273783, Sigma Aldrich, Dermstadt, Germany) at pH: 2.8 (solvent A), and acetonitrile (solvent B), (Pro. N. 34851, Sigma Aldrich, Dermstadt, Germany). The phenolic acids were detected at 260 nm and the gradient was programmed from 0% B to 35% B in 24 min with a 10 min isocratic held at 100% B. The flavonoids elution gradient was begun at 90% A and 10% B, and then ramped to 35% B in 27 min; this was followed by 100% eluent B for next 10 min; the UV/Vis detector wavelength was fixed at 350 nm.

Standards of gallic acid (Pro. N. G7384), caffeic acid (Pro. N. C0625), ferulic acid (Pro. N. 90034), p-coumaric acid (Pro. N. C9008), vanillic acid (Pro. N. 94770), sinapic acid (Pro. N. D7927), cinnamic acid (Pro. N. C80857), quercetin (Pro. N. Q4951), naringenin (Pro. N. 52186), naringenin chalcone (Pro. N. PHL83877), kaempferol (Pro. N. K0133), apigenin (Pro. N. 42251), and myricetin (Pro. N. 72576) were purchased from Sigma Aldrich, Dermstadt, Germany. The standard solutions were prepared by mixing phenolic acids (gallic, caffeic, ferulic, p-coumaric acid, vanillic, sinapic, and cinnamic acids) and flavonoids (quercetin, naringenin, naringenin chalcone, kaempferol, apigenin, and myricetin) to identify and quantify phenolic compounds in the TP extracts. For each sample, three injections were performed.

### 2.5. Antioxidant Activity Assay

Antioxidant activity was assessed by using the DPPH radical scavenging method, with a slight modification [20]. Briefly, 2 mL of DPPH (Prot. N. D9132, Sigma Aldrich, Dermstadt, Germany) solution in methanol (125 μM) were added to 50 μL of sample solutions at different concentrations. The decrease in absorbance at 517 nm was recorded by a spectrophotometer (Cary 60 UV-Vis, Agilent Technologies, Santa Clara, CA, USA) for 90 min at 10 min intervals. The results were expressed as IC_50_ i.e., the extract concentration that scavenges 50% of DPPH [21], which was compared with that of Trolox (Prot. N. 238813, Sigma Aldrich, Dermstadt, Germany) and ascorbic acid (Prot. N. PHR1008, Sigma Aldrich, Dermstadt, Germany) determined after 30 min of reaction at 517 nm.

### 2.6. Anti-Inflammatory Assay

#### 2.6.1. Cultivation of Caco-2 Cells

The epithelial cells from human intestinal (Caco-2 cells) were purchased from Public Health England (ECACC 86010202), Wiltshire, United Kingdom and they were cultivated in a 75 cm^2^ flasks by using 10% inactivated fetal bovine serum in Dulbecco’s Modified Eagle’s Medium (DMEM). Antibiotics penicillin (100 U mL^−1^), streptomycin (0.1 mg mL^−1^) and amino acid L-glutamine (2 mM) were added to the medium. The cells were grown in an incubator at 37 °C with a humidified atmosphere (95% air and 5% CO_2_). After the cells were at confluence, their transfer into multi-wells was done. 

Caco-2 cells were incubated for 2 h with IL-1β (20 ng mL^−1^) in the absence or presence of TP extracts to assess the anti-inflammatory effects of TP extracts. The alcoholic part was dried in a rotatory vapor until to obtain the residual powder that was successively dissolved in the DMEM prior to the assay. Positive experimental controls were cells only incubated with IL-1β, whereas the cell viability was measured by considering blank (untreated cells) [22].

#### 2.6.2. Cell Incubation

The experiments started on the 2nd day after the cell’s reached confluence. Before starting with the incubation assay, cell viability was measured, as described previously [22]. Caco-2 cells were then treated with different samples prepared in two different concentrations of total polyphenol content: 15 µg mL^−1^ and 25 µg ML^−1^ in the presence of IL-1β in the medium. Cells treated with IL-1β alone were used as the positive control, which allowed for setting 100% of inflammation induction in the experimental study. An untreated control sample is always needed to calculate the relative changes in gene expression in the sample of interest. The effects of the different molecules on inflammation will be indicated as changes in the expression of target genes that are related to the untreated control [23]. The treatment was performed in triplicate for each sample.

#### 2.6.3. Expression of IL-8 Measurement by Real-Time PCR Analysis (qPCR)

PCR amplification was performed in a total volume of 20 μL using iQ™ SYBR^®^ Green Supermix (170-8882, Bio-Rad Laboratories Inc., Hercules, CA, USA) and the following primers:

IL-8 (version 1, IL8-1) forward: 5′-CTG GCC GTG GCT CTC TTG GCA G-CCT TCC TG-3′; reverse: 5′-GGC AAC CCT ACA ACA GAC CCA CAC AAT A-CA-3′ (395 bp) [24].

IL-8 (version 2, IL8-2) forward: 5′-ATG ACT TCC AAG CTG GCC GTG GCT-3′; reverse: 5′-TCT CAG CCC TCT TCA AAA ACT TCTC-3′.

The GAPDH reference gene was amplified with the following primers: forward (nt 38-57) 5′-GGA AGG TGA AGG TCG GAG TC-3′; reverse (nt 218-237) 5′-CAC AAG CTT CCC GTT CTC AG-3′ which yield a 200 bp product [25].

Quantitative analysis was performed while using a MyiQ thermal cycler to quantify the SYBR Green with a dedicated proprietary software (Bio-Rad Laboratories, Inc., Hercules, CA, USA) and data were collected using MyiQ Real-Time PCR Detection System Software (Bio-Rad Laboratories, Inc., Hercules, CA, USA). The following program was used: 10 min at 95 °C, then 40 cycles of denaturation (20 s at 95 °C), annealing (30 s at 65 °C), and extension (30 s at 72 °C), at the end one last cycle at 65 °C. Each experiment was calibrated with the cDNA obtained from unstimulated Caco-2 cells, and a sample without cDNA was considered as negative control.

Relative amounts of target genes as compared to the GAPDH reference gene were calculated according to Livak’s method [24].

### 2.7. Statistical Analyses

The average and standard deviation values were calculated according to standard procedures and the results were analysed by the ANOVA bootstrap, Duncan test while using the SPSS 25 (IBM, New York, NY, USA). 

The Partial Least Square method (PLS) was applied to perform multiple linear regressions of IL-8 de-activation expression fold vs. A-PP concentration [26]. To do so, the variables were un-scaled and, by using SCAN software (Minitab Inc., State College, PA, USA), the cross-validation leave-one-out was chosen as methodology. An improvement of the goodness of fit coefficient—*R*^2^ and goodness of prediction coefficient—*R*^2^*cv* were considered as the criteria of the “removing variables” steps made to recognize the lesser number of independent variables (i.e., A-PP molecules) that are linked to anti-inflammatory effects (dependent variable). 

## 3. Results and Discussion

### 3.1. TP Chemical Characterization and Fermentation

TPs had very high moisture content that affected their storability (Table 1). From a chemical point of view, fibres (ADL + hemicellulose + cellulose) were the most abundant fractions; hemicellulose and cellulose came from peel, while ADL (i.e., the more recalcitrant fraction) was attributable, above all, to the lignin, cutin, and suberin of the seed coats [18,27]. The remaining fractions, which were described as CS, were composed by oil, protein, sugar, and organic acids, which are the more biodegradable compounds (Table 1) [27]. Short chain organic acids and ethanol were a relevant fraction of CS of TP for the degradative and fermentation processes in action (Table 1). Ethanol, lactate, and acetate were the products of the biological metabolism of lactic microorganisms (LAB) present in TP [14] (Table 2); pH level (6.86) and remaining fatty acids were typical of aerobic degradative metabolisms [28]. When fermentation started, the pH dropped immediately to very low values (pH < 4) because of the increase in lactic acid that reached the maximum concentration after 20 days of the process. In fact, lactic acid (pK_a_ of 3.86) contributed the most to the decline in pH during fermentation, because it is about 10 to 12 times stronger than the other major acids, such as acetic acid (pK_a_ of 4.75) and propionic acid (pK_a_ of 4.87). Other LAB metabolites (ethanol and acetate) remained almost constant; on the contrary, no-LAB acids were going to be consumed until only traces remained. A lactic acid/acetic acid ratio was applied as an indicator of LAB fermentation stability [28]; values of 2–3 meant that stable and optimal conditions were reached from the 20th day of the process; however, the prevalence of LAB was considered as a precautionary measure after 60–100 days when no-LAB metabolites become traces. LAB fermentation is extensively applied as a cheap method in the food preservation industries [29]. This effect comes about because of the very low pH and anti-microbial compounds production that influences the activity of membrane-bound enzymes and exo-enzymes. In addition, lactic acid is able to enter into the bacteria, lowering cellular pH and killing the microorganisms. Although the complete microorganism elimination occurred for pHs that were lower than 2.5, pHs around 3.5 were effective in eliminating several food-borne pathogens or enteric contaminants after some weeks of treatment [30]. 

The fermentation moderately decreased the TP organic matter content while a great effect occurred on macromolecular composition (Table 1); as expected, the relative content of CS, the easily biodegradable fraction, increased, and at the same time all fibers decreased. However, quantitative investigation confirmed the CS augmentation and hemicellulose and ADL consumption while no change occurred for cellulose. Pentose sugars that composed hemicellulose were ideal feedstocks for LAB metabolism; ADL had no defined chemical composition, but its LAB consumption was explainable while supposing that microbial activity changed the cell wall structure to make carbohydrates fractions available that were not usable before (e.g., pectin) [31].

### 3.2. TP Polyphenol Composition and Antioxidant Activity

TPC was applied to estimate the fraction of polyphenols in extracts (Table 3). Total polyphenols belong to a very abundant fraction that is closely-related to the vegetal fiber and a minor one chemically conjugated with small functional groups or free, i.e., the aglycone fraction A-PP [32]. Polyphenols and fiber had strong ester links that required hard acidic hydrolysis to be broken. On the other hand, soft extraction with water or alcohols was applied to extract the free or weakly linked fractions. The extraction conditions and solvents chosen were additional factors that affected the molecule recovery. Based on previous TP polyphenol characterization, methanol was the best solvent, being chosen to guarantee a very good extraction yield of the more concentrated molecules [33]. The TPC of TP1 and TP2 were almost similar (Table 3), which is in agreement with the literature, obtained by applying the same extraction conditions where the range of TPC was into the range 716–3516 mg gallic acid g^−1^ dry matter [12]. A more in-depth characterization of the aglycone-polyphenols (A-PP) fraction was carried out in order to better understand which parts of the polyphenols had been extracted, since they were very interesting for their high bioactivity (Table 3).

A-PP extracted belonging to the flavonoids and phenolic acids accounted for 70% and 30%, respectively; gallic acid, chlorogenic acid, and cinnamic acid were the more concentrated phenolic acids and naringenin chalcone and naringenin were the most abundant flavonoids (Table 3). The high naringenin chalcone concentration is due to its accumulation during the naringenin metabolic pathway [34].

Based on the HPLC fingerprint, the whole A-PP extracted content was calculated as the sum of phenol acids and flavonoids and compared with the corresponding TPC data (Table 3). A-PP for both TP1 and TP2 accounted for the greater part of TPC (83% TPC and 96% TPC, respectively, for the TP1 and TP2), thus suggesting that extraction conditions were able to obtain the more active polyphenols fraction.

Bioactivity was firstly measured as antioxidant power (Table 3) by using IC_50_ and compared with ascorbic acid (IC_50_ = 153 μg mL^−1^) and Trolox (IC_50_ = 100 μg mL^−1^) known to have high antioxidant capability. The TP1 had the lowest IC_50_, while TP2 was similar to Trolox, thus confirming that TP hydrophilic components have good antiradical activity (Table 3). 

Fermentation has been reported to be able to increase TPC and A-PP for the release from fiber of polyphenol fractions, although the results were not well in agreement based on the biomass, polyphenols typology, and fermentation method adopted [35,36].

In this work, TPC increased 17% and 12.5%, but A-PP significantly decreased during fermentation down to 57 ± 2% as an average of the starting value after 250 days (Table 3), in agreement with literature [37].

No significant A-PP flavonoids qualitative changes occurred between TP and TPF, since all of the molecules become less concentrated with the exception of the quercetin that increased [38]. Phenolic acids however remained constant and decreased for TP1F and TP2F for the reduction/conservation of most of the molecules with the exception of gallic and cinnamic acids, which increased. Similar behaviors were already described during sourdough LAB fermentation when a global A-PP reduction occurred, while fiber-ferulic acid bound hydrolysis improved the recovery of that phenolic acid [36].

TPF showed a significant difference between TPC and A-PP. The explanation is that, although TPC was usually associated with the presence of polyphenols, the Folin–Ciocalteu reagent was not specific for that class of molecules but rather for benzene derived substituents typical not only of polyphenols, but of other several molecules, such as amino acids, sugars, acids, etc. [36,39]. The extraction conditions and solvents chosen in addition affected the capability to extract some of the molecules. Since the TPC vs. A-PP difference occurred after fermentation we could suppose that aglycone fractions in the TPC were, in fact, LAB metabolism products. Aromatic organic acids, reducing sugars, and aromatic amino acid are probable constituents with cutin and suberin monomers. The augmentation of the difference after fermentation is, therefore, due to the production of non aglycone molecules. That fraction’s origin was supposed to have originated from polyphenols that were previously fiber-linked and then successively metabolized into de-glucosides, sulfoconjugates, glucoronides, and other forms [40].

Bioactivity increased with fermentation for both TPs (+26% and +42% for TP1F and TP2F respectively), nevertheless no significant correlation existed with TPC (*r* = 0.8, *p* < 0.2, *n* = 4). At the same time, A-PP had an opposite trend with respect to IC_50_, which means that, for TPF, the most part of the antioxidant activity was due to non A-PP. 

The explanation becomes more difficult when we consider that polyphenols have different antioxidant power for class, molecules, or derivate metabolites that affect the concentration vs. activity relationship [40]. The flavonoids were more active in comparison with the phenolic acids and A-PP were more bioactive than bound ones [40]. 

Thus the fraction of molecules defined as (TPC minus A-PP) were correlated with IC_50_ and a better result was found (*r* = 0.9, *p* < 0.1, *n* = 4) in comparison to that found for TPC vs. IC_50_. This result indicated that, effectively, TPC-A-PP contained an anti-oxidant fraction, but its presence was not sufficient to totally explain the anti-oxidant activity.

TPC accounted for 10–15% of the methanol-TP extract; therefore, the presence of other antioxidant molecules cannot to be excluded.

Vitamin C, for example, is present in tomato peel, bound to the cell-membrane structure that negatively affected its extractability; recovery was improved by LAB’s capability to break the links, so there was increased vitamin C release after fermentation.

The difficulty found in identifying the molecules that are responsible for the antioxidant effect is typical of what occurs when complex extracts are considered. This is due to the presence of several bioactive substances, since their co-existence generates numerous interactions. Potentiation, antagonism addition, and synergy are known effects that result in the final antioxidant activity deriving from chemical reactions, such as regeneration, spatial distribution, metal chelation, and mutual protection. The presence of pro-oxidant agents, the solubility of antioxidants in reaction media, and the solvent effects might reduce the overall activity [41].

The antioxidant bioactivity of TPF does better than that of raw ones thank to the production of fermentation metabolites that increase starting biomass nutritional value and potential health benefits. In addition, LAB fermentation is cheap and guarantees the biomass safety, all positive effects that renewed interest for its application. 

### 3.3. Anti-Inflammatory Properties 

Inflammation is a body stress status that is recognized to be the precursor of several diseases. Intestinal cells are able to respond to inflammatory signals by triggering various intracellular signal transduction cascades to control the expression of genes, including cytokines and chemokines, like IL-1, IL-6, IL-8, and TNF-a, due to their high exposure to inflammatory events. The human colon epithelial cell line Caco-2 secretes chemokine IL-8, which directs the migration of leukocytes, monocytes, and macrophages. The ability of polyphenol to influence IL-8 production has been used to evaluate the experimental anti-inflammatory properties. 

**Figure 1:**
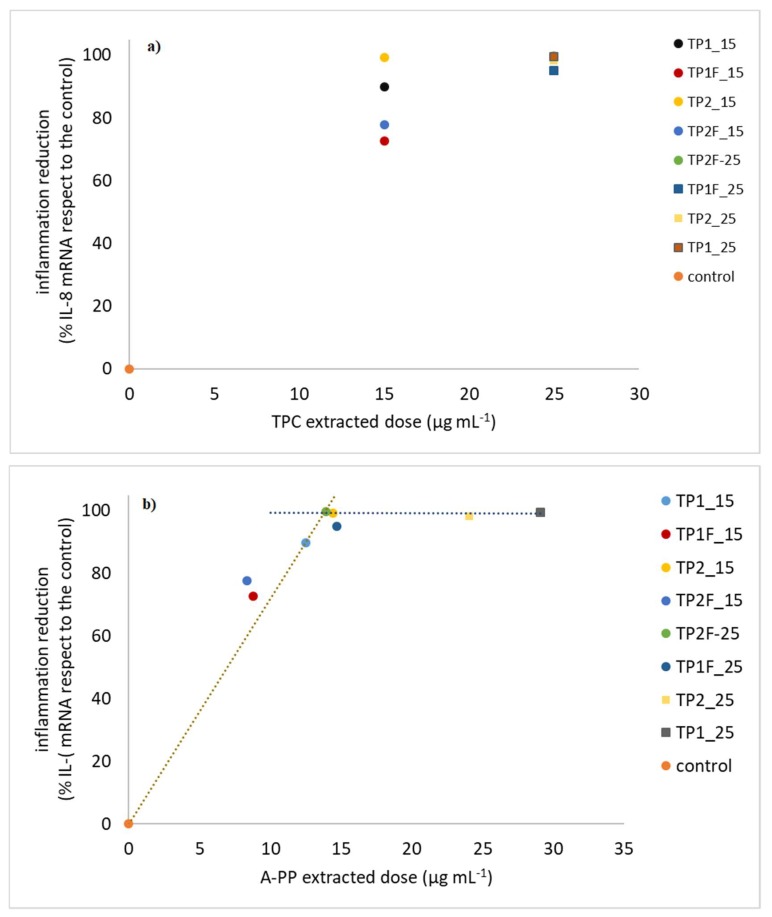
Relationships among inflammation reduction and TP extract dose expressed as total polyphenol content (**a**) and A-PP extracted (**b**).

TP and TPF were tested for their anti-inflammatory capability. The results indicated that all the TP extracts had very high anti-inflammatory effects, being described by a dose-dependent first linear phase followed by a plateau reached in correspondence with the complete inflammation elimination (Figure 1). 

TPC and A-PP doses were considered with the aim of identifying the TP extract fraction responsible for the effect. Dosing TPC = 15 μg mL^−1^ TPs were more bioactive than TPF, while the same effect was shown when considering TPC = 25 μg mL^−1^, since it corresponded to the plateau phase (Figure 1a,b). Minimum effective concentration (MEC) i.e., lowest dose that eliminated inflammation completely is a fundamental item of information for applicative purpose; by using the TPC = 15 μg mL^−1^ dose, it was found that TPF had anti-inflammatory activity of 78% in comparison with TP.

A-PP have been reported to be anti-inflammatory agents, being able to modify the expression of more pro-inflammatory genes, such as multiple cytokines, lipoxygenase, nitric oxide synthases, and cyclooxygenase, with particular reference to the regulation of the expression of NF-kB [42]. 

The doses were recalculated in order to correlate the anti-inflammatory effects of A-PP, resulting in being similar for TP but very different for TPF (Table 4). 

This dose expression positioned the most part of the samples into the dose-effect range and only TP1_25 and TP2_25 were at plateaus (Figure 1b). TP and TPF were now aligned to define together a linear straight phase (% reduction= (7.19 ± 0.31) * A-PP, *R*^2^ = 0.99, *p* < 0.001, *n* = 7) (Figure 1b); this result highlighted that LAB fermentation did not reduce anti-inflammatory capacity, but preserved the starting one and a common MEC of 13.7 μg mL^−1^ was calculated.

Further investigation led us to conclude that A-PP was responsible for the anti-inflammatory effect and LAB fermentation preserved that bioactivity. With reference to A-PP composition, it is worth noting that TP’s more concentrated flavonoids (naringenin and naringenin chalcone) were more effective than more concentrated phenolic acids (chlorogenic acid and cinnamic) for the capability to interact with different biological targets due to their different structure [43].

Naringenin inhibits TNF-α-induced TLR2 expression by inhibiting the nuclear factor-κB (NF-κB) and JNK pathways in adipocyte cells [44], whereas naringenin chalcone reduced the production of TNF-α and MCP-1 through IκB-α degradation in the RAW 264 macrophages that were stimulated by lipopolysaccharide [45].

Among phenolic acids, cinnamic acid and gallic acid inhibited NF-κB activation, in particular phosphorylation of IκB and NF-κB-dependent p65 acetylation, respectively; nevertheless, gallic acid inhibited the activation of COX-2 [43,46]. In particular, chlorogenic acid has shown inhibitory activity on the cytokine IL-8 production in the Caco-2 inflamed cells either through directly suppressing the NF-κB activation or indirectly via the inhibition of the upstream signaling pathways [47].

The combination of several bioactive substances has been described as enhancing the final anti-inflammatory effect for the capability to act against greater numbers of inflammatory mechanisms at the same time [35]. However, several chemical interactions affected the bioavailability of the molecules. The flavonoids co-existence improved their chemical stability and solubility, thus positively the bioavailability, but the same molecules competed with phenolic acid for cellular transportation, causing adsorption interference [41].

In vegetal extracts, several bioactives co-existed with others, often unknown, thus making it impossible to experimentally understand all of the chemical and biological interactions that contributed to the final bioactivity.

PLS was more reliable than other techniques when identifying relevant variables and their magnitudes of influence, especially in the cases of small sample size and low tolerance [48]. Coefficient importance was considered a discriminant parameter to select the variables. In this work, PLS was based on the identification of a dose of A-PP molecules vs. anti-inflammatory effect (expressed as IL-8 de-activation) relationship. At the end of several PLS cycles, the best Goodness-of-Fit and Goodness-of-Prediction of the regression model of *R*^2^ = 0.95 and *R*^2^*cv* = 0.62 were reached. 

Multiple PLS regression indicated that IL-8 inactivation was well explained by the naringenin chalcone, gallic acid, kaempferol, and apigenin having important coefficient values of 31%, 25%, 24%, 20% respectively. All of the molecules selected represented were the greater part of the A-PP (molecules selected 73 ± 15% of A-PP), and naringenin chalcone and gallic acid together accounted alone for 71 ± 15% of A-PP, therefore contributing the greatest amounts of the molecules’ dose to give bioactivity. On the other hand, from a qualitative point of view, their contribution corresponded to 56% of the importance, while kaempferol and apigenin had greater effects, taking their low concentration into consideration.

PLS selection confirmed the higher bioactivity of flavonoids with respect to phenolic acids (75% and 25% of the importance, respectively) [49]. 

The exclusion of other flavonoids more concentrated than naringenin that demonstrated higher concentration and the selection of kaempferol and apigenin suggested that PLS choice was not based only on the quantitative aspect, but also considered the action mechanism. Kaempferol for example was noted to be very potent phenolic compound for its capacity to affect anti-inflammatory by affecting two different inactivation pathways (inhibited STAT-1 and NF-kB) [50] on the contrary of the other molecules selected by PLS, where action was addressed to a single biological receptor. Similarly, apigenin has demonstrated strong anti-inflammatory properties via modulation of the gene expression of inflammatory cytokines via acting on the NF-κB and MAPK signaling pathways and through the pro-inflammatory mediators, such as cyclooxygenase, lipoxygenase, and nitric oxide synthases [51].

## 4. Conclusions

In conclusion, polyphenols from TPs in raw pomace or following fermentation can represent inexpensive and economic raw materials, and their use can decrease the waste discarding issues from the tomato processing industry. By exploiting the most favorable extraction methods, an improvement of the anti-oxidant properties was found for the production of bioactive substances during fermentation. Nevertheless, anti-inflammatory activity was preserved. That property, together with the good safety aspect of the fermented biomass, will suggest the employment of the extract for further application as supplementary elements or additive ingredients in nutraceutical, cosmetic and bioactive molecules industries. 

## Figures and Tables

**Table 1:** Tomato pomace (TP) macromolecular composition and evolution during fermentation.

Time Day	d.m.	VS	CS	Hemicellulose	Cellulose	ADL
	% Wet Weight	mg g^−1^ d.m.				
0	22.75 ± 0.01 b *^,^**	973.9 ± 6.4 b	155.9 ± 4.2 a	137.8 ± 3.7 b	250.1 ± 3.4 a	456.2 ± 3.8 c
1	22.72 ± 0.17 b	975.3 ± 5.8 b	161.5 ± 7.2 a	129.3 ± 4.1 ab	251.6 ± 1.4 a	457.6 ± 5.4 c
20	22.83 ± 0.07 b	973.4 ± 2.7 b	198.8 ± 9.2 ab	110 ± 5.8 a	250.8 ± 2.7 a	450.2 ± 2.1 c
30	22.97 ± 0.07 b	969.8 ± 5.8 b	212.3 ± 2.9 b	110.1 ± 6.8 a	253.7 ± 1.2 a	423.4 ± 1.5 b
60	21.7 ± 0.35 a	964.6 ± 3.8 ab	222.1 ± 3.2 b	111.8 ± 3.7 a	259.2 ± 4.3 a	407 ± 2.8 a
105	21.33 ± 0.41 a	962.8 ± 2.4 ab	221.7 ± 2.2 b	100.9 ± 1.2 a	273.4 ± 2.7 b	404.1 ± 1.7 a
240	21.36 ± 0.12 a	951.1 ± 1.1 a	221.6 ± 4.1 b	109 ± 3.3 a	270 ± 5.9 b	399.4 ± 1.8 a

Legend: d.m. dry matter; VS volatile solid; CS cell soluble; ADL acid detergent fibre * data are expressed as average data ± standard deviation of the TP1 and TP2 samples. ** data followed by the same letter in the same column are not statistically different (ANOVA Bootstrap, *p* < 0.05, post-test Duncan).

**Table 2:** pH, short chain fatty acids, and ethanol evolution during TP1 and TP2 fermentation.

Fermentation Time	pH	Ethanol	Formate	Acetate	Propionate	Lactate
Days		mg g^−1^ d.m.				
0	6.59 ± 0.07 b *^,^**	8.04 ± 3.22 a	2.53 ± 0.63 a	10.47 ± 6.64 a	1.63 ± 0.65 a	28 ± 2 a
1	3.86 ± 0.03 a	12.84 ± 3.47 ab	8.89 ± 0.33 ab	20.21 ± 9.08 ab	4.29 ± 2.47 a	25 ± 2 a
20	3.84 ± 0.01 a	15.67 ± 0.68 b	12.48 ± 0.11 c	36.78 ± 1.18 c	4.62 ± 3.14 a	86 ± 1 d
30	3.88 ± 0.01 a	14.13 ± 1.43 b	7.55 ± 0.44 b	33.94 ± 2.04 c	5.15 ± 5.21 a	65 ± 2 c
60	3.76 ± 0.01 a	15.75 ± 0.69 b	4.51 ± 0.23 ab	36.87 ± 0.86 c	5.65 ± 3.64 a	65 ± 2 c
105	3.89 ± 0.01 a	14 ± 0 b	6.1 ± 1.12 ab	34.6 ± 0.91 c	4.89 ± 1.18 a	65 ± 1 c
240	3.79 ± 0.02 a	14.51 ± 0.72 b	3.75 ± 0.22 a	36.26 ± 3.92 c	5.37 ± 4.44 a	67 ± 4 c

* Data are expressed as average data ± standard deviation of the TP1 and TP2 samples. ** data followed by the same letter in the same column are not statistically different (ANOVA Bootstrap, *p* < 0.05, post-test Duncan).

**Table 3:** Total phenolic content (TPC), aglycone-polyphenols (A-PP) polyphenols extracted content, and antioxidant activity of TP and TPF.

Parameter		Measure Unit	TP1	TP1F	TP2	TP2F
	TPC	µg GA g^−1^ d.m. TP	2300 ± 120 a*A**	2700 ± 40 b	3200 ± 90 aB	3600 ± 170 b
	IC_50_-DPPH	µg mL^−1^	57.9 ± 1.8 aA	75.7 ± 3.8 b	92.7 ± 0.6 cB	131.1 ± 0.7 d
Phenolic acids extracted	gallic acid	μg g^−1^ d.m	146.5 ± 4.3 aA***	249.3 ± 0.4 b	222.7 ± 15.1 aB	185.7 ± 27 a
	chlorogenic acid		89.2 ± 6.2 aA	99.4 ± 7.5 a	271.7 ± 8.5 aB	73.5 ± 14 a
	vanillic acid		10.3 ± 0.6 bA	7.4 ± 0.6 a	1.1 ± 0.2 aA	2.6 ± 0.7 b
	caffeic acid		94.4 ± 2.8 bA	19.2 ± 2.1 a	61.6 ± 2.1 bB	8.7 ± 0.8 a
	ferulic acid		8 ± 1 bA	4.5 ± 0 a	22.1 ± 1.4 aA	9.3 ± 1.9 a
	P-coumaric acid		22.7 ± 0.9 bA	11 ± 1.2 a	64.6 ± 8.9 bB	11.7 ± 5.2 a
	sinapic acid		23.8 ± 4.3 bA	3.3 ± 0.9 a	2.5 ± 1.7 bA	2.2 ± 1.1 a
	cinnamic acid		105.6 ± 3.7 aA	171.1 ± 14 b	88.9 ± 22.7 aA	164 ± 5.3 b
	Sum		*500*	*565*	*735*	*458*
Flavonoids extracted	quercetin		37.3 ± 0.8 aA	70.3 ± 6.1 b	52.7 ± 2.4 aB	77.9 ± 0.6 a
	naringenin		298 ± 9 bA	49.6 ± 1.9 a	659 ± 7 bB	328 ± 3 a
	naringenin chalcone		1016.9 ± 5.9 bA	873.4 ± 0.5 a	1260.5 ± 17.7 bB	1081.6 ±12.8 a
	apigenin		32.4 ± 4.2 bA	10.6 ± 0.2 a	66.4 ± 0.7 bB	38.8 ± 2.4 a
	myrecetin		2.3 ± 0.6 bA	0 a	2.5 ± 0.2 bA	0 a
	kaempferol		32.5 ± 4.1 bA	15.7 ± 0.1 a	0 ± 0 aB	23.5 ± 1.5 b
	Sum		*1419.4*	*1019.6*	*2341.1*	*1549.8*
A-PP extracted phenolic content	phenolic acids + flavonoids		1919.4	1584.6	3076.1	2007.8

* Data followed by the small letter in the same line are not statistically different (ANOVA Bootstrap, *p* < 0.05, post-test Duncan) ** data followed by the same capital letter in the same line for the same TP are not statistically different (ANOVA Bootstrap, *p* < 0.05, post-test Duncan) *** molecules identified and quantified corresponded to the most part of the whole spectra (68.25%, 78.64%, 85.65%, and 77.52% of the TP1, TP1F, TP2, and TP2F spectra, respectively).

**Table 4:** Effect of TP extracts on IL-8 release from Caco-2 cells.

Samples	Dose		Inflammation
	µg TPC mL^−1^	µg A-PP Extracted mL^−1^	IL-8 m-RNA Expression
TP1	15	12.52	2.53 ± 0.15
	25	29.09	0.13 ± 0.01
TP1F	15	8.80	7.1 ± 0.08
	25	14.67	1.31 ± 0.06
TP2	15	14.42	0.26 ± 0.01
	25	24.03	0.45 ± 0.01
TP2F	15	8.37	5.68 ± 0.08
	25	13.94	0.06 ± 0.03
Control	0	0	26.84 ± 0.13
